# Response Characteristics of Nitrifying Bacteria and Archaea Community Involved in Nitrogen Removal and Bioelectricity Generation in Integrated Tidal Flow Constructed Wetland-Microbial Fuel Cell

**DOI:** 10.3389/fmicb.2020.01385

**Published:** 2020-06-23

**Authors:** Longmian Wang, Qingqing Pang, Fuquan Peng, Aiguo Zhang, Ying Zhou, Jianjun Lian, Yimin Zhang, Fei Yang, Yueming Zhu, Chengcheng Ding, Xiang Zhu, Yiping Li, Yibin Cui

**Affiliations:** ^1^Nanjing Institute of Environmental Sciences, Ministry of Ecology and Environment, Nanjing, China; ^2^College of Environment, Hohai University, Nanjing, China; ^3^College of Energy and Environment, Anhui University of Technology, Ma’anshan, China

**Keywords:** nitrification, tidal flow constructed wetland, microbial fuel cell, microbial community structure, electricity generation

## Abstract

This study explores nitrogen removal performance, bioelectricity generation, and the response of microbial community in two novel tidal flow constructed wetland-microbial fuel cells (TFCW-MFCs) when treating synthetic wastewater under two different chemical oxygen demand/total nitrogen (COD/TN, or simplified as C/N) ratios (10:1 and 5:1). The results showed that they achieved high and stable COD, NH_4_^+^-N, and TN removal efficiencies. Besides, TN removal rate of TFCW-MFC was increased by 5–10% compared with that of traditional CW-MFC. Molecular biological analysis revealed that during the stabilization period, a low C/N ratio remarkably promoted diversities of ammonia-oxidizing archaea (AOA) and ammonia-oxidizing bacteria (AOB) in the cathode layer, whereas a high one enhanced the richness of nitrite-oxidizing bacteria (NOB) in each medium; the dominant genera in AOA, AOB, and NOB were *Candidatus Nitrosotenuis*, *Nitrosomonas*, and *Nitrobacter*. Moreover, a high C/N ratio facilitated the growth of *Nitrosomonas*, while it inhibited the growth of *Candidatus Nitrosotenuis*. The distribution of microbial community structures in NOB was separated by space rather than time or C/N ratio, except for *Nitrobacter*. This is caused by the differences of pH, dissolved oxygen (DO), and nitrogen concentration. The response of microbial community characteristics to nitrogen transformations and bioelectricity generation demonstrated that TN concentration is significantly negatively correlated with AOA-shannon, AOA-chao, 16S rRNA V4−V5-shannon, and 16S rRNA V4−V5-chao, particularly due to the crucial functions of *Nitrosopumilus*, *Planctomyces*, and *Aquicella*. Additionally, voltage output was primarily influenced by microorganisms in the genera of *Nitrosopumilus, Nitrosospira*, *Altererythrobacter*, *Gemmata*, and *Aquicella.* This study not only presents an applicable tool to treat high nitrogen-containing wastewater, but also provides a theoretical basis for the use of TFCW-MFC and the regulation of microbial community in nitrogen removal and electricity production.

## Introduction

Earth’s biogeochemical cycles have been largely affected by human activities (Yu et al., 2019), especially by a large amount of nitrogen released from agriculture and animal husbandry, which is the root of many environmental issues such as eutrophication and drinking water pollution. To this end, various methods have been purposed to remove nitrogen. However, these traditional nitrogen removal approaches (i.e., activated sludge process, trickling filter, and oxidation pond) have some limitations, such as low removal rate or high operating cost (Tanwar et al., 2008). It is acknowledged that total nitrogen (TN) in wastewater mainly consists of ammonia (NH_4_^+^-N), nitrite (NO_2_^–^-N), and nitrate (NO_3_^–^-N). Generally, nitrification, denitrification, and anaerobic ammonia oxidation (anammox) processes are the typical biological pathways used for nitrogen elimination (Zhi et al., 2015). Also, removal of nitrogen in wastewater has been investigated by biological method, and/or its combination with natural and ecological approaches (Wu et al., 2017). With two prominent challenges nowadyas, i.e., energy scarcity and non-point sourced nitrogen, finding economical and sustainable nitrogen removal technologies is particularly difficult.

Constructed wetlands (CWs) have been extensively designed and studied as a sustainable technology to treat water bodies contaminated with low-concentration nitrogen (Zhi et al., 2015). However, many CWs only limitedly reduce nitrogen levels with fluctuating results; thus, an improved treatment method is urgently needed (Liu et al., 2015). Vertical flow constructed wetlands (VFCWs) and horizontal flow constructed wetlands (HFCWs) are the initial designs of modified constructed wetlands. Due to low TN removal efficiency of a single-stage CW, some multi-stage treatment systems, such as HF-VFCWs or VF-HFCWs, have also been developed (Vymazal, 2007, 2013). However, these systems are practically difficult to use due to complex operation conditions and a huge demand for land.

Recently, an expanded system consisting of a microbial fuel cell (MFC) coupled to a CW (CW-MFC) is becoming increasingly popular because it allows simultaneous wastewater treatment and electricity generation (Yadav et al., 2012; Doherty et al., 2015). CW and MFC share the redox gradient between an anaerobic anode and an aerobic cathode, in addition to electricity produced via MFC; thus, they have enhanced removal efficiencies (Srivastava et al., 2015). Tidal flow constructed wetland (TFCW), operated through a rhythmic sequential cycle of a “feeding/flooding” phase and a “draining/resting” phase, has been proposed as a compact and efficient method that can enhance the nitrogen removal (Zhi and Ji, 2014). However, the nitrogen removal pathway and the underlying mechanism that governs the nitrogen transformation process of TFCW-MFC are not clearly understood.

Generally, nitrification is a biological oxidation of ammonium to nitrite and nitrate, and is the first and most crucial step in TN removal. It is conducted in two sequential steps via several microorganisms that are phylogenetically distinct, including ammonia-oxidizing archaea (AOA), ammonia-oxidizing bacteria (AOB), and nitrite-oxidizing bacteria (NOB) (Zhang et al., 2011). Oxidation of NH_4_^+^-N is considered mainly driven by chemolithoautotrophic AOB (Guimaraes et al., 2017). However, a recent study has shown that AOA, which is ubiquitously distributed in aquatic environments, often has higher abundance than AOB, and its abundance is correlated with nitrification rates (Leininger et al., 2006). On the other hand, oxidation of NO_2_^–^-N (to NO_3_^–^-N) depends mainly on NOB (Zhang et al., 2019). The quantitative relationships between ammonia oxidation rate and nitrogen functional genes (AOA and AOB) in TFCW have been analyzed (Li et al., 2015). However, the accuracy of AOA, AOB, and NOB compositions in TFCW-MFC remains unconfirmed, and the interactions between the dominant genera and environmental factors have yet to be established; these have limited our ability to optimize the treatment processes. Influent chemical oxygen demand/total nitrogen (COD/TN; generally simplified as C/N) ratio has been considered as a key factor influencing the nitrogen removal in CWs (Meng et al., 2018). It is also an important parameter affecting the activity of AOB and NOB (Li et al., 2017). The recommended optimal influent C/N ratio for the removal of nitrogen (from approximately 80% TN) by CW-MFC is 5.37 (Wang et al., 2019). Unfortunately, to date, related research conducted using TFCW-MFC has not been reported.

To fill this knowledge gap, two laboratory-scale TFCW-MFCs were developed, and their nitrogen removal performance and bioelectricity generation from synthetic wastewater under two different C/N ratios were examined. The spatiotemporal variations of the diversity and community structure of AOA, AOB, NOB, and 16S rRNA V4−V5 region at filters and electrodes were investigated using the high-throughput sequencing. The relationships between the genetic characteristics of the four types of microorganisms (i.e., diversity and relative abundance of dominant genera) and effluent indexes [i.e., pH, dissolved oxygen (DO), temperature (T), NH_4_^+^-N, NO_3_^–^-N, NO_2_^–^-N, and TN] were also determined. This work provides comprehensive theoretical support for improving TN removal through the optimization of main functional microbial composition and populations in TFCW-MFC.

## Materials and Methods

### Device Configuration

Two identical integrated TFCW-MFCs, namely Device A (influent C/N = 10:1) and Device B (influent C/N = 5:1), were constructed in the Laboratory of Watershed Water Environment at the Nanjing Institute of Environmental Sciences, Nanjing, China. Each TFCW-MFC was covered with a black adhesive tape and was placed at room temperature (22°C). The main structure of TFCW-MFC was made from a polyacrylic plastic cylinder (internal diameter = 50 cm, height = 80 cm). Five layers were constructed from the bottom to the top including: a support layer made of cobble (diameter = 15–30 mm) with a depth of 15 cm; a quartz sand II layer (diameter = 5–10 mm) with a depth of 25 cm; an anode layer with a depth of 10 cm; a quartz sand I layer (diameter = 5–10 mm) with a depth of 25 cm; and a cathode layer with a depth of 5 cm. The anode and cathode layers were constructed using granular activated carbon (GAC; specific surface area = 450–800 m^2^/g, diameter = 2–5 mm; Jiangsu Zhuxi Activated Carbon Co., Ltd., Jiangsu, China) and stainless-steel mesh (0.3-mm thickness, 1.7-mm diameter; Nanjing Zhongdong Chemical Glass Instrument Co., Ltd., Nanjing, China), respectively. The anode and cathode layers were connected by a 1-mm titanium wire connecting to a 1000-Ω external resistance. *Cattail* (Typha latifolia L.), as the wetland plant, was planted in the cathode layer, which would be combined with plant roots to promote oxygen transport to create better aerobic conditions (Oodally et al., 2019). Four sampling ports (each with a diameter of 40 mm) arranged in the cathode, quartz sand I, anode, and quartz sand II layers, were used to collect filters or electrodes, so as to analyze the characteristics of the related bacteria.

### Inoculation and Operation of TFCW-MFC

Prior to the operation, the TFCW-MFC device was inoculated (at the anode compartment) with conventional activated sludge (1.1 L) obtained from the Jiang Xinzhou wastewater treatment plant (Nanjing, China). Sodium acetate (1.0 g/L) was mixed with the supernatant at a ratio of 1:1, and the mixture was pumped continuously into the bottom of the system at a flow rate of 0.02 m^3^/d. Fifty percent of the effluent was pumped back to the device to enrich the electrogens in the anode layer. After 1 month of operation, the maximum voltages were found stable, indicating that TFCW-MFC has been successfully started. Ten strains of *cattail* planted for 1 month at an ambient temperature were transferred to the cathode layer during the start-up of TFCW-MFC.

**FIGURE 1:**
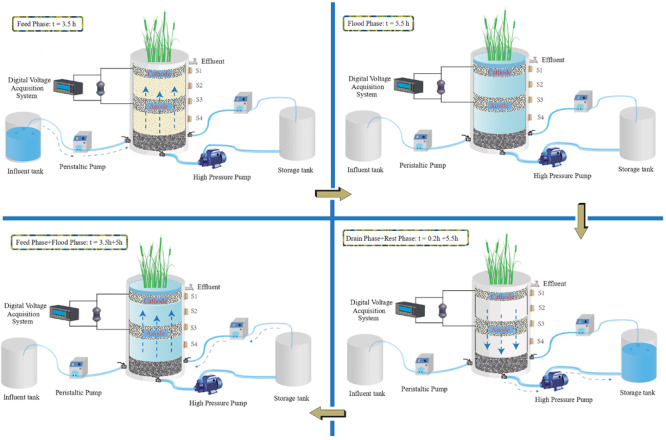
Schematic diagram of TFCW-MFC during a typical cycle mode.

After the system was stable, two TFCW-MFCs were operated with two different C/N ratios, and synthetic wastewater was then introduced into the systems, which were processed for another 90 days (Supplementary Table S1). Figure 1 shows the schematic diagram of the TFCW-MFC operation. At the beginning, 40 L of synthetic wastewater was fed into the bottom of TFCW-MFC using a peristaltic pump (BT100-1L, Baoding Longer Precision Pump Co., Ltd., Baoding, China). After a 5-h flooding period, the treated wastewater was drained from the bottom of the devise into the water storage tank for 12 min using a high pressure pump; afterward, the TFCW-MFC was rested for up to 4 h. Thereafter, the treated wastewater in water storage tank was pumped back into TFCW-MFC about 2 h in the bottom-up flow mode. After submerging 5 h, the wastewater was discharged from the bottom of the device as a full cycle accomplishment. The complete hydraulic retention of this system including recirculation was 18.4 h. Water samples were collected from the influent tank and the total water outlet once every 2 days and then analyzed.

### Sample Collection and Analysis

Samples of influents and effluents were collected for wastewater treatment analysis every 2 days using a 250-mL beaker. COD, NH_4_^+^-N, NO_2_^–^-N, NO_3_^–^-N were detected by the closed reflux titrimetric method, Nessler’s reagent colorimetric method, colorimetric method and ultraviolet spectrophotometric screening method, respectively, according to the Standard Methods (American Public Health Association [APHA], 2005). TN was determined by spectrophotometry with alkaline potassium persulfate ultraviolet. DO, pH, and temperature were measured using a portable multiparameter water quality monitoring (HQ40d, HACH, United States). The voltage was monitored through a Digital Voltage acquisition system (Keithley 2700, Tektronix, United States) and was recorded once per minute. The internal resistance was determined and calculated from the polarization curves, and the power density curve of CW-MFC was calculated according to Eqs. (1, 2) (Logan, 2008).

(1) $$J=\frac{U}{\mathrm{SR}}$$

(2) $$P=\frac{U^{2}}{\mathrm{SR}}$$

where J is the mean current density (mA/m^2^), P is the power density (mW/m^2^), S is the surface area of the anode electrode (m^2^), U is the voltage (V), and R is the external resistance (Ω).

### High-Throughput Sequencing Analysis

Microorganism samples were collected from the four substrate-sampling ports at days 30 and 90 in Table 1. DNA extraction was performed using 0.25 g soil by the Fast DNA spin kit for soil (MO BIO Laboratories, Carlsbad, CA, United States) following the manufacturer’s protocol. The extraction was taken from each obtained DNA for agarose gel electrophoresis (1.2%) testing. High-quality DNAs with clear bright bands (approximate 30 ng/μL DNA content) could be used as templates for polymerase chain reaction (PCR). AOA, AOB, NOB, and V4−V5 region of 16S rRNA were amplified by PCR using universal microbial primers (Supplementary Table S2). The PCR amplification condition was as follows: initial denaturation at 90–95°C for 2 min; 30–35 cycles of denaturation at 90–95°C for 30 s, annealing at 50–60°C for 30 s, and extension at 70–75°C for 30 s; and final extension at 70–75°C for 5 min. The sequences with a similarity threshold of 97% were clustered into operational taxonomic units (OTUs). The sequencing was performed using an Illumina Miseq (2 × 300) platform by the Personal Biotechnology Co., Ltd. (Shanghai, China).

**TABLE 1:** Locations of different sampling points in TFCW-MFC.

**Day**	**C/N**	**S1**	**S2**	**S3**	**S4**
30	10:1	Ac1	Au1	Aa1	Ab1
	5:1	Bc1	Bu1	Ba1	Bb1
90	10:1	Ac2	Au2	Aa2	Ab2
	5:1	Bc2	Bu2	Ba2	Bb2

*A − represents the devices with influent C/N = 10:1; B − represents the devices with influent C/N = 5:1; c, u, a, b mean cathode layer, upper layer, anode layer, bottom layer, respectively; (1) operation on the 30th day; (2) operation on the 90th day.*

Alpha diversity indexes (including Chao and Shannon indexes) were calculated based on the OTU numbers using quantitative insights into microbial ecology (QIIME) (Caporaso et al., 2010). The Chao indexes were mainly used to reflect the species richness between samples. The larger the index value, the more abundant the species; The Shannon indexes mainly reflected the diversity of the communities in the samples. The larger the Shannon index, the richer the diversity. The difference between microbial communities was analyzed based on the phylogenetic information using the Weighted UniFrac (Wang et al., 2017). The relationships between microbial communities were determined based on the distance matrix using the principal coordinates analysis (PCoA). Analysis of environmental factors that had the most effects on the microbial community structure was conducted using the redundancy analysis (RDA) by Canoco software (version 4.5) (Zhang et al., 2019). The impact of each environmental factor on the microorganism was analyzed based on the RDA data using the variation partitioning analysis (VPA) by Canoco.

### Statistical Analysis

Data analyses were conducted using Microsoft Excel 2010, and the figures were prepared using Origin 2016. Means among all treatment groups were compared by one-way analysis of variance (ANOVA) using SPSS 19.0 software package. Based on the assumption of normal distribution, the Pearson correlation, which was used to determine the relationships between alpha diversity at the genus level and effluent nitrogen concentration, and between nitrogen removal and the relative abundance of the dominant genera of each of the four types of microorganisms in 16 sample sizes, was also calculated using SPSS 19.0. *P* < 0.05 and *P* < 0.01 indicate the significant differences. In addition, the *P*-values for multiple testing remain uncorrected, including the False Discovery Rate procedure, due to the small number of samples in this study.

## Results and Discussion

### Wastewater Treatment Performance

**FIGURE 2:**
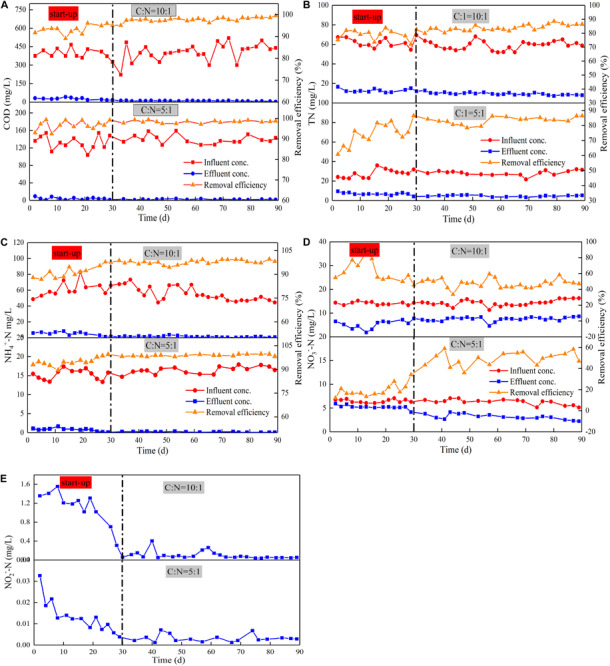
Average removal efficiency and change in concentrations of COD **(A)**, TN **(B)**, NH_4_^+^-N **(C)**, NO_3_^–^ -N **(D)**, NO_2_^—^N, **(E)** with various C/N ratios.

Device A and Device B were stable after 30 days of operation, and they achieved COD removal efficiencies of 97.66 and 98.36%, respectively, during the stabilization period (Figure 2A). The obtained COD removal efficiencies were higher than previous studies of the CW-MFC to treat livestock sewage, which have typical removal efficiencies ranging from 71 to 81% (Zhao et al., 2013; Doherty et al., 2015; Liu et al., 2020). The COD removal load of Device A was 40.26 g/m^2^⋅d, which was higher than that reported previously (9.8 g/m^2^⋅d by Albuquerque et al. (2009), 29.20 g/m^2^⋅d by Konnerup et al. (2009). This enhanced COD removal efficiency is likely attributed to the intensified oxygen supply generated by the tidal flow operation (Oon et al., 2017). The effluent DO in both Device A (5.31 mg/L) and Device B (4.83 mg/L) were higher than the influent DO, which may be due to the oxygen intake from the atmosphere via employing the tidal flow mode. Meanwhile, the direct reduction by heterotrophic and electroactive microorganisms in the root area and plant uptake in the system also improved COD degradation rates (Yang et al., 2020). Additionally, wastewater treatment by the coupled MFC has been reported to be more effective than that by CWs. Compared with traditional CWs, the biodegradation of organic compounds is enhanced by the transfer of electrons from the anode to the cathode. Study has also shown that CW-MFC is superior to conventional CW concerning the COD removal rate (Wang et al., 2016). Therefore, it is highly likely that high-efficient removal of COD by TFCW is due to the integration of MFC.

As shown in Figure 2, the removal of nitrogen other than NO_3_^–^-N in Device B was stable after 30 days of operation, which indicates that the microorganisms used in nitrogen removal are mature. The average removal rate of TN (82.39%) and NO_3_^–^-N (52.16%) in Device A were higher than those in Device B (79.25% of TN, 36.72% of NO_3_^–^-N). By contrast, the removal rates of NH_4_^+^-N in both devices were similar with values of higher than 95% (95.94% in Device A, 97.13% in Device B). The removal rates of NH_4_^+^-N and TN were similar, inferring that the removal of TN may be in the form of NH_4_^+^-N and a certain amount of the effluent NO_3_^–^-N may be accumulated. This indicates that the loss of carbon source may lead to incomplete denitrification, thus causing the accumulation of NO_3_^–^-N. The TN removal rate of TFCW-MFC was higher than that of CW-MFC previously reported (74% has been reported by Wang et al., 2017, 69% has been reported by Wu et al., 2017; Table 2). This observation further demonstrated that the developed device has higher TN removal efficiency, which may be caused by the use of tidal flow. It could increase the abundance of nitrogen-transformation genes. The coenrichment of these genes is attributed to their environmental adaptions to the balance condition of nitrification and denitrification via oxygen transfer, as well as their related distinct ecological niches (Zhi et al., 2015). Thus, the increase in gene abundance is more likely to be the reason accounting for the enhanced denitrifying activity. Furthermore, better TN removal rates (90% and 87%) in Table 2 were obtained using CW-MFC in latest reports (Srivastava et al., 2020; Tao et al., 2020). Compared with the results in the literature, the advantage of this study was that the efficient TN removal could be achieved under relatively low C/N ratios and short retention time. However, the NO_3_^–^-N removal rate is low because high DO is not conducive to denitrifying microorganism growth. Although the concentration differences of NO_2_^–^-N in effluents between Device A and B were found, the NO_2_^–^-N removal rates in both systems were more than 90%. Since NO_2_^–^-N is in the intermediate valence state of nitrogen transformation and it has an unstable characteristic, the impact of C/N ratio on NO_2_^–^-N attenuation could be negligible. Nonetheless, exorbitant carbon source concentration inhibits denitrification instead of boosting it (Liu et al., 2012). Thus, the C/N ratio should be further optimized to achieve higher NO_3_^–^-N removal rate.

**TABLE 2:** Operating conditions and performance comparison of TFCW-MFC and other CW-MFCs.

**Type**	**Liquid volume (L)**	**Wastewater type**	**Influent COD (mg/L)**	**C/N**	**Hydraulic retention time (d)**	**COD removal rate (%)**	**TN removal rate (%)**	**Max. power**	**References**
Vertical upflow	3.7	Swine	1058	7.25	0.9−1.25	76.5	49.7	9.4 mW/m^2^	Zhao et al., 2013
Horizontal flow	96	Synthetic	250	−	3.2	80−100	−	0.15 mW/m^2^	Villaseñor et al., 2013
Vertical flow	1.8	Synthetic	770−887	−	1	90.9	−	43.63 mW/m^3^	Srivastava et al., 2015
Upflow−downflow	8.1	Swine	583	9.25	1	64	58	0.28 W/m^3^	Doherty et al., 2015
Horizontal subsurface flow	115	Domestic wastewater	323	7.8	0.17	61	−	36 mW/m^2^	Corbella et al., 2015
Vertical downflow	−	Synthetic	813	10	4	57.4	−	8.08 mW/m^2^	Wang et al., 2016
Vertical downflow	−	Synthetic	207.3	3.77	2	80	74	21.53 mW/m^2^	Wang et al., 2017
Vertical upflow	−	Synthetic	646	−	1	98	−	184.75 mW/m^3^	Oon et al., 2017
Vertical upflow	3.5	Swine	647	4.72	40	69	69	112 mW/m^2^	Wu et al., 2017
Vertical upflow	−	Synthetic	−	−	3	82.32	82.46	3.71 W/m^2^	Xu et al., 2018
Vertical flow	−	Synthetic	730	6.4	−	97	−	229 mW/m^3^	Oodally et al., 2019
Downflow−upflow	11.5	Swine	900	3	2	88.07	−	496.4 mW/m^3^	Liu et al., 2020
Horizontalflow−vertical upflow	2.85	Synthetic	880	22	0.63	99.5	90	25 mW/m^3^	Srivastava et al., 2020
Vertical upflow	−	Municipal wastewater treatment plants effluents	−	5.4	2	64.04	88.78	6.09 mW/m^2^	Tao et al., 2020
Tidal flow	40	Synthetic	523.11	10	0.77	97.66	82.39	25.78 mW/m^2^	This study
Tidal flow	40	Synthetic	114.05	5	0.77	98.36%	79.25	16.97 mW/m^2^	This study

### Electricity Generation

**FIGURE 3:**
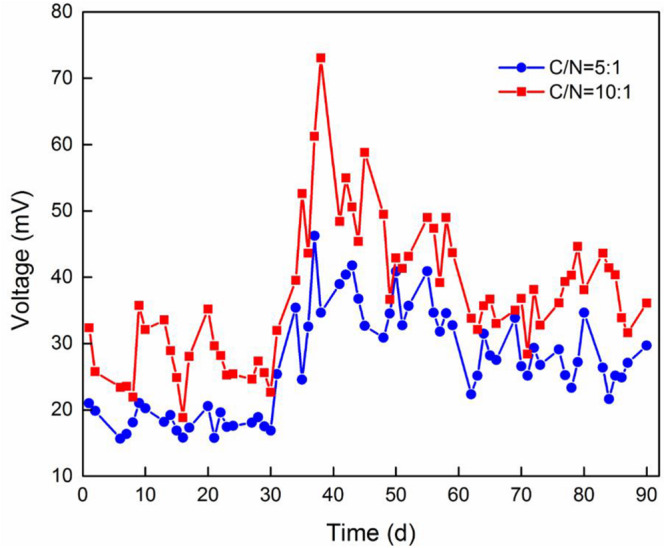
Voltage output of two TFCW-MFC devices operated with various C/N ratios.

The voltage curve of TFCW-MFC under different influent C/N ratios is shown in Figure 3. According to the curve, Device A and Device B had the average voltages of 40.25 and 26.85 mV, respectively. Moreover, the maximum power densities of the system operated with C/N ratios of 10:1 and 5:1 were 25.78 and 16.97 mW/m^2^, respectively, while the current densities were 0.18 and 0.11 A/m^2^, respectively, and the internal resistances were 151 and 174 Ω, respectively. This clearly indicated that low internal resistance causes the increase in power density. Similar and high power density values have been reported by previous studies in Table 2: Wang et al. (2017) have reported a value of 21.53 mW/m^2^ in CW-MFC planted with *T. orientalis*; Corbella et al. (2015) have reported a maximum value of 36 mW/m^2^, which was harvested by implementing MFC in HFCW during the treatment of effluent from a hydrolytic up-flow sludge blanket reactor. Low power density obtained by TFCW-MFC may be attributed to the following three reasons: (1) a small ratio of electrode surface to CW-MFC system volume may obstruct the accumulation of electrochemically active microorganisms on the electrode; (2) electrons may be largely depleted due to high concentrations of electron acceptors (NO_2_^–^-N, NO_3_^–^-N, oxygen, etc.) presented in the biofilter devices (Wang et al., 2016); and (3) spacing between anode and cathode electrodes (25 cm) may be too large, as has been described by Oon et al. (2017), who observed that the voltage output in up-flow constructed wetland-MFC tends to decrease when the spacing between anode and cathode electrodes are increased from 15 to 30 cm. Hiegemann et al. (2017) have also reported that the voltage output increases with the increase of organic concentration, as the voltage output is dependent on the electrons and protons transferred from carbon sources at the anode. However, excessive organic loading is not conducive to electricity generation because the multiplication of methanogens can inhibit the power generation (Martin et al., 2010), or organics may not be completely oxidized at the anode, thereby generating an anaerobic environment that causes the CW-MFC to have decreased power output or even stop working (Villaseñor et al., 2013). It is worth noting that the voltage output of both devices increased after 30 days of operation, but thereafter gradually decreased until it became stable. This indicated that high electrogenic microbial activity or organic concentration can promote electricity generation in the system. After 40 days of operation, the denitrifying microorganism in the system may become more abundant than the electrogenic microorganism; consequently, the electricity-producing ability was inhibited.

### Diversity of Microbial Community

**FIGURE 4:**
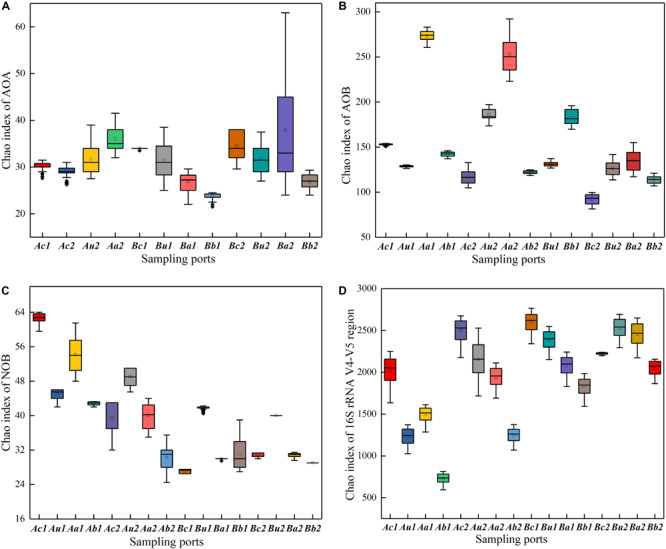
Relative change of Chao indexes of panels **(A)** AOA, **(B)** AOB, **(C)** NOB, and **(D)** 16S rRNA V4–V5 region, at different sampling points of TFCW-MFCs. The horizontal line in the box represents the median. The size of the box represents the data dispersion (Interquartile range = IQR).

The Chao index was used to evaluate the richness of microbial community structure. As shown in Figures 4A–D, the Chao indexes of the two systems decreased in the following order: 16S rRNA V4−V5 region > AOB > NOB > AOA. Interestingly, AOB had a higher abundance than AOA, indicating that the microenvironment of the filter is more suitable for AOB growth. While one study has reported that AOA in many marine and terrestrial ecosystems is more abundant by one to two orders of magnitude than AOB (Mincer et al., 2007); by contrast, another study has indicated that AOB is more dominant (Liu et al., 2014). This inconsistent result may be primarily related to the high-strength NH_4_^+^-N at the influent, beneficial to AOB rather than AOA reproduction. Furthermore, the spatial distribution of each type of microorganisms is different and it changes over time, due to the collaborative functions of nitrogen concentration, pH, and temperature (Fierer et al., 2009; Hallin et al., 2009; Fan et al., 2011; Zhang et al., 2019). The microbial richness in the upper layer of V4−V5 region was higher than that in the lower layer, which may mainly be due to the aerobic microsites adjacent to plant roots and exudates from rhizomes, where the oxygen released from the *Cattail*’s root into the cathode could promote the growth of microorganisms. The richness of NOB in each substrate layer in Device A was higher than that in Device B (Figure 4C), indicating that a higher C/N ratio is beneficial to the survival of NOB. The richness of AOA and AOB were higher in the anode layer on the 90th day than that on the 30th day, due to that the exoelectrogenic microorganism and AOA/AOB gradually adapted to coexist on the anode in a facultative oxygen environment (DO = 0.9–1.5 mg/L at the anode in Supplementary Figure S1). This result is consistent with the observation by Xu et al. (2018), who have reported that the microbial richness in the anode is the highest in the CW-MFC system.

As shown in Supplementary Figure S2 and Supplementary Tables S3–S6, the diversity of microorganisms in the system became unstable after 30 days, and the C/N ratio had a greater influence on the space-time distribution of all four types of microorganisms in the same layer, especially Ab1 (1.42) and Bb1 (3.1) within AOB, Aa1 (2.53), Ba1 (1.75) within NOB, and Ab1 (3.56) and Bb1 (5.58) within the V4−V5 region. It is possible that at the beginning of the operation, carbon and nitrogen concentrations may have a direct or indirect effect on the microorganism in the anode and the bottom layers of the two devices. In contrast, the microbial community structure was relatively stable after 90 days, and the lower influent C/N ratio (5:1) appeared to facilitate the growth of AOA and AOB in the cathode layer, as well as that of all types of microorganisms in the V4−V5 region. The microorganism in the cathode layer is more diverse, which is likely due to the higher DO (Wu et al., 2011; Supplementary Figure S1) caused by the reoxygenation ability of the tidal flow, or there may be a large number of aerobic electrogenic microorganisms in AOA and AOB. The influent C/N ratio had little effect on the diversity of microorganisms in the same layer.

### Comparative Analysis of Microbial Community Structure

**FIGURE 5:**
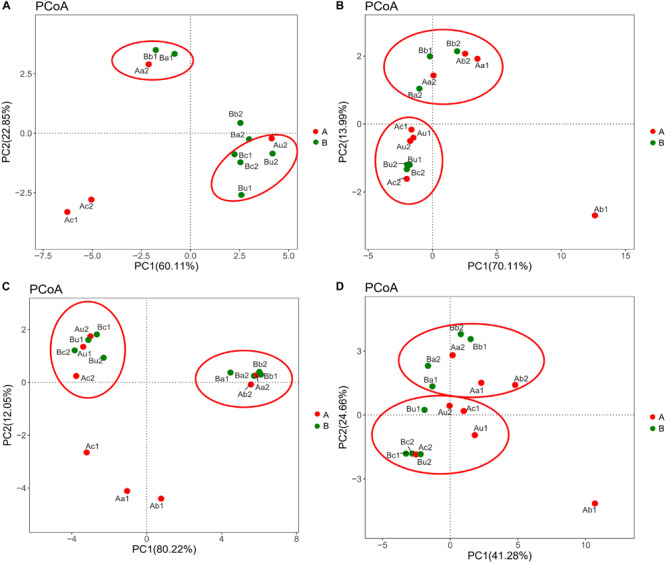
Difference in distribution of microbial community of panels **(A)** AOA, **(B)** AOB, **(C)** NOB, and **(D)** 16S rRNA V4–V5 region in different samples in the two devices. Data were obtained based on the phylogenetic lineages using the weighted Fast UniFrac PCoA. The red ellipse indicates that different samples with similar microbial community structure are clustered together.

**FIGURE 6:**
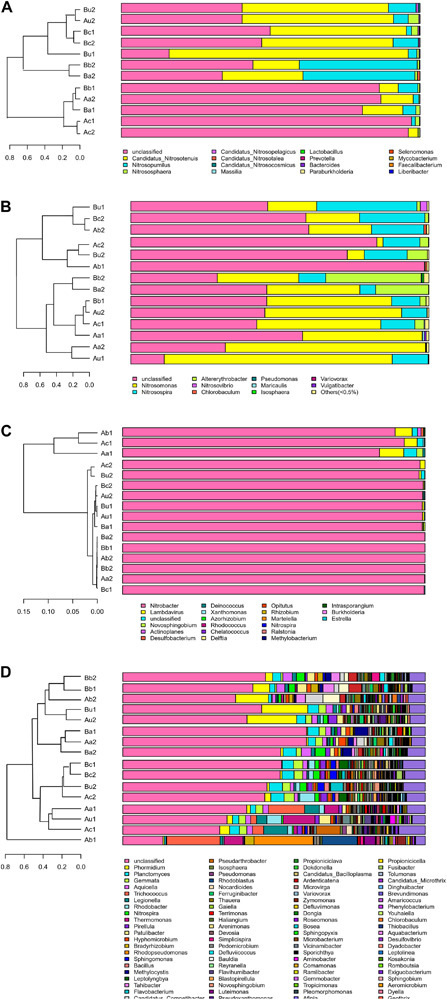
Bar plot and cluster tree of microbial community of panels **(A)** AOA, **(B)** AOB, **(C)** NOB, and **(D)** 16S rRNA V4–V5 region.

The differences in microbial community structure of the two systems were evaluated based on the phylogenetic lineages by the weighted Fast UniFrac PCoA. As shown in Figure 5, there was a certain loss of AOA in Device A and of AOB in device B, which is likely to be caused by the instability of the system at the initial operation of the tidal flow, and the oxygen concentration in the lower layer of the device may be insufficient. Additionally, the microbial community structure of AOA, AOB, NOB, and V4−V5 region were changed by 82.96%, 84.1%, 92.27%, and 65.94%, respectively. These results indicated that the microbial community structure is largely separated by space, which is consistent with the bar plot and cluster tree shown in Figure 6. The C/N ratio also had influence on the structure of the four types of microorganisms. While that of *Nitrobacter*, which belongs to NOB, was not affected by the C/N ratio, the relative abundances of *Candidatus Nitrosotenuis* (belongs to AOA) and *Nitrosomonas* (belongs to AOB) in the cathode layer were significantly promoted by a low C/N ratio (5:1) during the stabilization period. This result is consistent with the influence of C/N ratio on the diversity of the microbial community, indicating that these predominant genera may cause the change of diversity. Further, as shown in Figure 6, a low C/N ratio (5:1) significantly increased the relative abundance of *Nitrosopumilus* (belongs to AOA) in all layers. AOA was found to participate in ammonia removal processes; however, its functions and contributions in the processes under the influence of MFC remain unclear. These results show that at a low C/N ratio (5:1), the cathode layer is beneficial to the survival of the dominant archaea AOA. On the other hand, a high C/N ratio (10:1) promoted the relative abundance of *Nitrosomonas* (belongs to AOB) in the anode and the upper layer during the stabilization period. This may be caused by the increased production of carbon dioxide in the anode layer, and AOB may consume a large amount of inorganic nitrogen while immobilizing carbon dioxide (He et al., 2016). These data indicate that the C/N ratio has a significant effect on the spatial distribution of the dominant bacteria AOB. The results further showed that a low C/N ratio is more suitable for the growth of *Candidatus Nitrosotenuis* (belongs to AOA), whereas a high C/N ratio is beneficial to the growth of *Nitrosomonas* (belongs to AOB). It is likely that *Nitrosomonas* can better adapt to a highly polluted environment than *Candidatus Nitrosotenuis*, as has been described by a previous study (Fan et al., 2011).

As shown in Figure 6C, the relative abundance of *Nitrobacter* in each sample was the highest (from 86.95 to 99.99%). This is consistent with the previous study, which has described that *Nitrobacter* is the single abundant genus in NOB (Zhang et al., 2019). However, it is opposite to another study using laboratory-scale devices, which has reported that *Nitrospira* is the predominant genus in NOB (Sayess et al., 2013). The difference is likely due to that *Nitrospira* can better adapt to low nitrite and oxygen concentrations, and *Nitrobacter* can outcompete *Nitrospira* at high substrate concentration (Maixner et al., 2006). The genera in the V4−V5 region was more abundant than those in other microorganisms, and the dominant microorganisms included *Phormidium*, *Planctomyces*, *Gemmata*, and *Aquicella* (Figure 6D). *Trichococcus* and *Rhodopseudomonas* preferred a high C/N ratio, while *Planctomyces* and *Gemmata*, which are *anammox* bacteria (Xu et al., 2018), were less affected by the C/N ratio. Although the discovery of anammox genera, the role of NH_4_^+^-N removal via anaerobic ammonium oxidation in TFCW-MFC was negligible in the presence of relatively high DO throughout the tidal flow.

### Correlation Between Single or Multiple Environmental Variables and Microbial Community Structure

**FIGURE 7:**
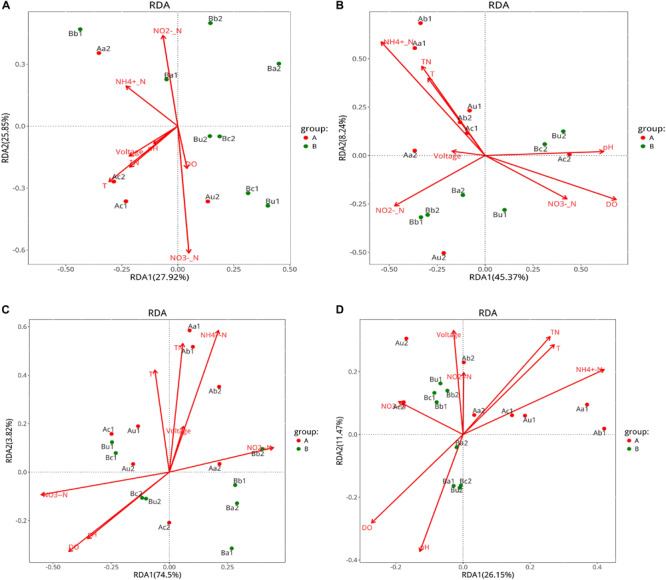
RDA analysis curves of panels AOA **(A)**, AOB **(B)**, NOB **(C)**, and 16S rRNA V4-V5 region **(D)**. Symbols indicate the samples and arrows represent environmental variables. Group A represents Device A operated with a C/N ratio = 10:1, and Group B represents Device B operated with a C/N ratio = 5:1. Environmental variables were chosen based on the significance determined from individual RDA results.

The effects of environmental variables, including nitrogen concentration, pH, DO, temperature, and voltage, on microbial community structure were analyzed using VPA (Supplementary Figure S3) and RDA (Figure 7). According to the VPA diagram (Supplementary Figure S3), the following results were observed; (1) AOA community structure was explained by the general chemical indexes (pH and DO, 29%), and temperature (21%); (2) AOB community structure was explained by the nitrogen concentration (12%), and the shared fraction between pH, DO, and nitrogen concentration accounted for 16%; (3) NOB community structure was explained by the shared fraction between pH, DO, and nitrogen concentration (37%), and the shared fraction between nitrogen concentration and temperature accounted for 33%; and (4) the community structure of V4−V5 region was explained by nitrogen concentration (14%), and the shared fraction between pH, DO, and nitrogen concentration accounted for 12%. These data were subjected to the RAD analysis to determine the correlation between specific environmental factors and the community structure of the four types of microorganisms under the influence of different C/N ratios.

The RDA results indicated that the residual concentration of NO_2_^–^-N is positively correlated with AOA, AOB, NOB, and microorganisms in the V4−V5 region in the lower layer of the two devices. Additionally, the residual concentration of TN and NH_4_^+^-N, and temperature were positively correlated with AOB, NOB, and the microorganisms in the V4−V5 region of Device A, but not with AOA. Moreover, the pH, DO, and residual concentration of NO_3_^–^-N were positively correlated with AOA, AOB, and NOB in the upper layer of the two devices at day 90. This is consistent with the results reported by Zhou et al. (2016), who have observed that multiple physicochemical parameters are involved in the relative abundance of nitrifying microorganisms grown in the same environment. Combined with the VPA results, we may conclude that the ecological niche of TFCW-MFC device is mainly influenced by pH, which serves as the crucial factor that changes the diversities and compositions of all four microbial communities. It appears that each microbial species has its own optimal survival environment; for example, the optimal conditions for *Nitrosomonas* (belong to AOB) are 35°C and pH 8.1 and those for *Nitrobacter* (belongs to NOB) are 38°C and pH 7.9 (Grunditz and Dalhammar, 2011). At day 90, the effluent pH and temperature in Device A were 7.47 and 23.5°C, respectively, while those in Device B were 7.34 and 23.1°C, respectively. The second most influential factor is DO, which plays an important role as an electron acceptor for nitrifying microorganisms; thus, it can determine microbial growth and reproductive rate (Yuan and Blackall, 2002). DO is also a key factor affecting the positive correlation between NO_3_^–^-N and microorganisms in the upper layer, and NO_2_^–^-N and microorganisms in the lower layer. AOB population size has been reported to increase with increasing soil NH_4_^+^-N concentration (Okano et al., 2004), and AOB and NOB growths rely on the energy provided by NH_4_^+^-N and NO_2_^–^-N (Winkler et al., 2012). However, the amount of AOA is not correlated with ammonia concentration (Di et al., 2009), therefore the effects of NH_4_^+^-N concentration on AOA, AOB and NOB are different. By considering the effects of voltage on microbial community, voltage had a greater impact on the V4−V5 region microorganisms than AOA, AOB, and NOB; it was also positively correlated with the V4−V5 region microorganisms in Device A only at day 90 and in Device B only at day 30. The V4−V5 region comprises many exoelectrogenic microorganisms (Corbella et al., 2015); it is likely that electrons are largely depleted due to the relatively high concentrations of electron acceptors such as NO_2_^–^-N, NO_3_^–^-N, oxygen, etc., Wang et al. (2016).

### Pearson Correlation Between Microbial Characteristic Parameters, Nitrogen Removal, and Voltage Output

To further study the effect of microorganisms on nitrogen removal and bioelectricity generation, the relationship between alpha diversity index, voltage output, and effluent nitrogen concentration was analyzed by the Pearson correlation. As shown in Table 3, TN concentration was significantly negatively correlated with V4−V5-chao and AOA-shannon with *R*-values of −0.646 and −0.693, respectively (*P* < 0.01), as well as with AOA-chao and V4−V5-shannon with *R*-values of −0.608 and −0.596, respectively (*P* < 0.05). This is mainly due to the dominant genera of AOA and V4−V5 region. The Pearson correlation analysis was employed to further clarify the correlation between the dominant microorganisms and nitrogen removal. The analysis was performed using the high-abundance microorganism only the genus with >10% of the total genus composition), and the effluent nitrogen concentrations from the devices using each of these microorganisms were then compared. As shown in Table 4, TN concentration was significantly negatively correlated with *Nitrosopumilus* (belongs to AOA) and *Aquicella* (belongs to V4−V5 region) with *R*-values of −0.529 and −0.572, respectively (*P* < 0.05), as well as with *Planctomyces* (V4−V5 region) with *R*-value of −0.666 (*P* < 0.01). *Nitrosopumilus* is a dominant genus of AOA, and a low C/N ratio could significantly promote its relative abundance in all layers during the stabilization period. Therefore, *Nitrosopumilus*, which was especially rich in anode and cathode layers, could accelerate the nitrification reaction and greatly promote the conversion of nitrogen. *Planctomyces* (V4−V5 region) belongs to the anammox bacteria that can use NH_4_^+^-N as an electron donor and NO_3_^–^-N/NO_2_^–^-N as an electron acceptor to convert nitrogen compounds in water into nitrogen. The correlation between the alpha diversity of each genus and NH_4_^+^-N is similar to that between the alpha diversity of genus and TN. Concentration of NH_4_^+^-N was significantly negatively correlated with V4−V5-chao, V4−V5-shannon, AOA-chao, and AOA-shannon with *R* = −0.826, −0.744, −0.688, and −0.746, respectively (*P* < 0.01), as well as with NOB-chao with *R* = −0.543 (*P* < 0.05). By contrast, it was significantly positively correlated with TN concentration with *R* = 0.828 (*P* < 0.01). The correlation between the genus and nitrogen removal (Table 4) further indicated that NH_4_^+^-N concentration was significantly negatively correlated with *Candidatus Nitrosotenuis* (belongs AOA), *Nitrobacter* (belongs NOB), and *Planctomyces* (belongs to V4−V5 region microorganisms) with *R*-values of −0.617 (*P* < 0.05), −0.648 (*P* < 0.01), and −0.819 (*P* < 0.01), respectively. *Candidatus Nitrosotenuis* was the dominant genus of AOA, and a low C/N ratio could significantly promote its relative abundance in the cathode layer during the stabilization period; thus, this genus is more conducive to the conversion of NH_4_^+^-N. Furthermore, as the most abundant genus of NOB, *Nitrobacter* is suitable for NH_4_^+^-N removal in wastewater, which has also been confirmed by our previous study (Zhang et al., 2019).

**TABLE 3:** Pearson correlation between alpha diversity at the genus level, voltage, and effluent nitrogen concentration (*n* = 16).

	**AOA−chao**	**AOB−chao**	**NOB-chao**	**V4−V5-chao**	**AOA-shannon**	**AOB-shannon**	**NOB-shannon**	**V4−V5-shannon**	**TN**	**NH_4_^+^-N**	**NO_3_^–^-N**	**NO_2_^–^-N**
AOB-chao	−0.154^a^											
NOB-chao	−0.316	0.682**										
V4-V5-chao	0.839**	−0.371	−0.379									
AOA-shannon	0.872**	−0.297	−0.433	0.821**								
AOB-shannon	−0.150	0.812**	0.522*	−0.333	−0.274							
NOB-shannon	−0.293	0.146	0.383	−0.173	−0.264	0.117						
V4-V5-shannon	0.721**	−0.325	−0.341	0.927**	0.747**	−0.264	−0.185					
TN	−0.608*	0.477	0.460	−0.646**	−0.693**	0.220	0.175	−0.596*				
NH_4_^+^-N	−0.688**	−0.413	−0.543*	−0.826**	−0.746**	0.296	0.096	−0.744**	0.828**			
NO_3_^–^-N	0.265	−0.232	0.233	0.392	0.234	−0.123	0.457	0.303	−0.169	−0.552*		
NO_2_^–^-N	−0.122	0.213	−0.188	−0.181	−0.005	0.542*	−0.424	−0.212	−0.179	0.013	−0.550*	
Voltage	0.740	−0.402	−0.639	0.825	0.477	−0.085	−0.244	0.758	−0.819	−0.775	0.951*	0.757

^*a*^coefficient of association (*R*); * significant correlation (*P* < 0.05); ** significant correlation (*P* < 0.01). Bold values mean significance correlation including p < 0.05 and p < 0.01.

**TABLE 4:** Pearson correlation between dominant genera of each of the four types of microorganisms, voltage, and effluent nitrogen concentration (*n* = 16).

	***Candidatus_ Nitrosotenuis***	***Nitro sopumilus***	***Nitro sosphaera***	***Nitro somonas***	***Nitro sospira***	***Alterery throbacter***	***Nitro bacter***	***Phor midium***	***Planc tomyces***	***Gem mata***	***Aqui cella***	**TN**	**NH_4_^+^-N**	**NO_3_^–^-N**	**NO_2_^–^-N**
*Nitrosopumilus*	0.123^a^	1													
*Nitrososphaera*	0.367	0.032	1												
*Candidatus*_ *Nitrosopelagicus*	0.791**	0.026	0.233												
*Nitrosomonas*	−0.273	−0.008	−0.017	1											
*Nitrosospira*	0.520*	−0.034	−0.185	−0.063	1										
*Altererythrobacter*	0.008	0.938**	−0.040	−0.025	−0.014	1									
*Nitrobacter*	0.368	0.306	0.236	0.029	0.331	0.219	1								
*Bradyrhizobium*	−0.387	0.198	−0.310	−0.231	−0.262	0.212	−0.115								
*Phormidium*	0.442	−0.158	0.423	0.122	0.559*	−0.174	0.219	1							
*Planctomyces*	0.552*	0.300	0.293	−0.308	0.233	0.218	0.399	−0.139	1						
*Sphingomonas*	0.478	0.075	−0.066	−0.378	0.316	0.068	0.230	−0.316	0.629**						
*Gemmata*	0.370	−0.273	0.212	−0.047	0.117	−0.290	0.047	−0.176	0.737**	1					
*Aquicella*	0.164	0.727**	0.221	0.289	0.118	0.653**	0.313	0.152	0.289	−0.100	1				
TN	−0.439	−0.529*	−0.177	0.186	−0.179	−0.422	−0.424	0.220	−0.666**	−0.234	−0.572*	1			
NH_4_^+^-N	−0.617*	−0.353	−0.415	0.263	−0.401	−0.287	−0.648**	−0.109	−0.819**	−0.443	−0.446	0.828**	1		
NO_3_^–^-N	0.527*	−0.292	0.566*	−0.018	0.418	−0.263	0.200	0.436	0.463	0.614*	−0.040	−0.169	−0.552*	1	
NO_2_^–^-N	−0.340	0.525*	−0.101	0.215	−0.103	0.579*	0.131	0.028	−0.384	−0.698**	0.575*	−0.179	0.013	−0.450	1
Voltage	0.900	0.983*	0.464	−0.326	0.963*	0.963*	0.439	−0.064	0.854	−0.999**	0.962*	−0.819	−0.775	0.951*	0.757

^*a*^coefficient of association (*R*); * significant correlation (*P* < 0.05); ** significant correlation (*P* < 0.01). The selected genus accounts for >10% of the total composition of each gene. Bold values mean significance correlation including p < 0.05 and p < 0.01.

The effluent concentration of NO_2_^–^-N was significantly positively correlated with AOB-shannon with *R* = 0.542 (*P* < 0.05), while may be related with the dominant genus *Altererythrobacter* (*R* = 0.579; *P* < 0.05). Additionally, NO_2_^–^-N concentration was also significantly positively correlated with *Nitrosopumilus* and *Aquicella* with *R*-values of 0.525 and 0.575, respectively (*P* < 0.05), while was negatively correlated with *Gemmata* with *R*-value of −0.698 (*P* < 0.01). NO_3_^–^-N concentration was significantly positively correlated with *Candidatus Nitrosotenuis*, *Nitrososphaera*, and *Gemmata* with *R* = 0.527, 0.566, and 0.614, respectively (*P* < 0.05). This demonstrated that the abundance of ammonia oxidizing bacteria increases with increasing effluent NO_2_^–^-N and NO_3_^–^-N concentrations. *Gemmata* belongs to anammox bacteria, which can convert ammonia nitrogen using NO_2_^–^-N as an electron acceptor; thus a negative correlation between NO_2_^–^-N and *Gemmata* was observed. Furthermore, voltage was significantly positively correlated with the effluent NO_3_^–^-N concentration, *Nitrosopumilus*, *Nitrosospira*, *Altererythrobacter* and *Aquicella* with *R* = 0.951, 0.983, 0.963, 0.963, and 0.962 (*P* < 0.05), respectively, while was significantly negatively correlated with *Gemmata* (*R* = −0.999; *P* < 0.01). These data indicate the above genera and the electrogenic bacteria in the system may work synergistically so that the effect of the electrogenic bacteria is promoted, causing the voltage output to increase. On the other hand, organic matter can be oxidized by electrogens at the anode of MFC, and the produced electrons can flow to the cathode by an external circuit (Puig et al., 2012). Consequently, NH_4_^+^-N loses its electrons and is converted to NO_2_^–^-N and NO_3_^–^-N by the action of microorganisms, as indicated by a higher abundance and diversity of the microorganism at the cathode and anode layers. *Gemmata*, which belongs to the phylum *Planctomycetes*, contains fibrillar nucleoid surrounded by electron-dense granules (Fuerst and Richard, 1991) that may have an adverse effect on the electrogenic microorganism in the system. In spite of these data, the distribution characteristics of the electrogenic microorganisms and their contribution to the electricity production have not been analyzed. Thus, studies on the topics should be further conducted.

## Conclusion

In summary, we demonstrated that the removal of COD, NH_4_^+^-N, and TN from wastewater with different C/N ratios (10:1 and 5:1) using TFCW-MFC were not significantly different. Compared to the conventional CW-MFC, our TFCW-MFC achieved a high and stable TN removal efficiency (77–89%) from wastewater containing various C/N ratios. Moreover, the high-throughput sequencing analysis confirmed that a low C/N led to enhanced AOA and AOB diversities in the cathode layer during the stabilization period, causing the community structure of the dominant genera to change drastically. A low C/N ratio (5:1) significantly promoted the relative abundance of *Candidatus Nitrosotenuis*, *Nitrosopumilus* (both belong to AOA) and *Nitrosomonas* (belongs to AOB) in the cathode layer during the stabilization period. On the other hand, a high C/N ratio (10:1) promoted the relative abundance of *Nitrosomonas* (belongs to AOB) in anode and upper layers. We further observed that multiple environmental variables collaboratively contributed to change of microbial compositions; for instance, pH, DO, and nitrogen concentration could potentially change the spatial distribution of the microbial community structure. The results further revealed that effluent nitrogen concentration could be reduced through changes of certain alpha diversities indexes of microorganisms or relative abundances of the dominant genera. We also found that high voltage output in two devices can be achieved by adjusting the distribution of dominant genera in the medium. Nonetheless, to understand whether or not anammox bacteria in TFCW-MFC can be used for a different NH_4_^+^-N removal pathway, further experiments should be performed.

## Data Availability Statement

The datasets generated for this study are available on request to the corresponding author.

## Author Contributions

LW and QP contributed to acquisition and analysis of data, drafted and revised the manuscript. YinZ and AZ made contributions to conception and design of the study. FP, FY, YimZ, CD, and XZ conducted the experiments and coordinated to acquire the data. YueZ and YL modified the pictures and tables throughout the manuscript. JL and YC provided the improvement suggestions, administrated, and supervised the project. All of the authors read and approved the final manuscript.

## Conflict of Interest

The authors declare that the research was conducted in the absence of any commercial or financial relationships that could be construed as a potential conflict of interest.
