# Supplementary material for: Response Characteristics of Nitrifying Bacteria and Archaea Community Involved in Nitrogen Removal and Bioelectricity Generation in Integrated Tidal Flow Constructed Wetland-Microbial Fuel Cell
Source: Front Microbiol. 2020 Jun 23;11:1385. doi: 10.3389/fmicb.2020.01385 (PMC7324634; doi:10.3389/fmicb.2020.01385)
Supplement: Supplementary file 1 [file Data_Sheet_1.doc]

*Supplementary Information for*

Response Characteristics of Nitrifying Bacteria and Archaea Community Involved in Nitrogen Removal and Bioelectricity Generation in Integrated Tidal Flow Constructed Wetland-Microbial Fuel Cell

**SUMMARY**

**Table S1** Water quality parameters of wastewater with different influent C/N ratios.

**Table S2** Sequence of specific primers used in high-throughput sequencing.

**Table S3** Diversity index of each clone library of AOA at different heights and time in TFCW-MFC.

**Table S4** Diversity index of each clone library of AOB at different heights and time in TFCW-MFC.

**Table S5** Diversity index of each clone library of NOB at different heights and time in TFCW-MFC.

**Table S6** Diversity index of each clone library of 16S rRNA V4-V5 region at different heights and time in TFCW-MFC.

**Fig. S1** DO profile of TFCW-MFC at different C/N ratios.

**Fig. S2** Relative change of shannon indexes at different sampling points of TFCW-MFCs.

**Fig. S3** VPA analysis curves of AOA (A), AOB (B), NOB (C), and 16S rRNA V4-V5 region (D).

**Table S1** Water quality parameters of synthetic wastewater with different influent C/N ratios.

| Parameters | C:N=10:1 | | C:N=5:1 | |
| --- | --- | --- | --- | --- |
|  | Average | Range | Average | Range |
| COD(mg/L) | 523.11 | 409.76-557.19 | 114.05 | 117.08-144.63 |
| TN(mg/L) | 57.53 | 53.55-62.28 | 25.41 | 23.84-36.07 |
| NH_4_^+^-N(mg/L) | 51.82 | 42.58-60.97 | 15.86 | 13.85-17.97 |
| NO_3_^-^-N(mg/L) | 15.17 | 13.84-17.88 | 6.63 | 5.76-7.61 |
| NO_2_^-^-N(mg/L) | 6.11 | 5.12-7.53 | 1.87 | 1.43-2.38 |
| pH | 7.25 | 7.02-8.08 | 7.18 | 6.84-7.73 |
| DO(mg/L) | 2.32 | 1.80-2.84 | 1.96 | 1.63-2.29 |

**Table S2** Sequence of specific primers used in high-throughput sequencing.

| Target | Specific primer sequence | References |
| --- | --- | --- |
| AOA | Arch amoA F 5'-STAATGGTCTGGCTTAGACG-3' | (Zhang et al., 2012) |
|  | Arch amoA R 5'-GCGGCCATCCATCTGTATGT-3' |  |
| AOB | amoA-1F 5'-GGGGTTTCTACTGGTGGT-3' | (Rotthauwe et al., 1997) |
|  | amoA-2R 5'-CCCCTCKGSAAAGCCTTCTTC-3' |  |
| NOB | NOB R 5'-TTTTTTGAGATTTGCTAG-3' | (Martins et al., 2011) |
|  | NOB F 5'-CTAAAACTCAAAGGAATTGA-3' |  |
|  | nxrA R 5’-TCYACAAGGAACGGAAGGTC-3’ |  |
|  | PolR 5'-ATSGCCATCATYTCRCCGGA-3' |  |
|  | MLR 5'-TTCATTGCRTAGTTWGGRTAGTT-3' |  |
|  | AMDGR 5'- CCCAACTATCCCTATTAATCAT-3' |  |
| 16S V4-V5 | 515F 5'-GTGCCAGCMGCCGCGGTAA-3' | (Xiao et al., 2018) |
|  | 926R 5'-CCGTCAATTCMTTTGAGTTT-3' |  |

Xiao, L.L., Liu, F.H., Liu, J.C., Li, J.J., Zhang, Y.C., Yu, J.F., Wang, O.M., (2017). Nano-Fe_3_O_4_ particles accelerating electromethanogenesis on an hour-long timescale in wetland soil. Environ. Sci.:Nano. doi:10.1039/C7EN00577F

Martins, G., Terada, A., Ribeiro, D.C., Corral, A.M., Brito, A.G., Smets, B.F., Nogueira, R., (2011). Structure and activity of lacustrine sediment bacteria involved in nutrient and iron cycles. Fems Microbiol. Ecol. 77, 666-679.

Rotthauwe, J.H., Witzel, K.P., Liesack, W., (1997). The ammonia monooxygenase structural gene amoA as a functional marker: molecular fine-scale analysis of natural ammonia-oxidizing populations. Appl. Environ. Microbiol. 63, 4704-4712.

Zhang, L.M., Hu, H.W., Shen, J.P., He, J.Z., (2012). Ammonia-oxidizing archaea have more important role than ammonia-oxidizing bacteria in ammonia oxidation of strongly acidic soils. Isme J. 6, 1032-1045.

**Table S3 Diversity index of each clone library of AOA at different heights and time in TFCW-MFC.**

| Sample | Sequences | OTUs | Chao | Shannon |
| --- | --- | --- | --- | --- |
| Ac1 | 47278 | 30 | 31 | 1.08 |
| Ac2 | 47018 | 29 | 30 | 0.74 |
| Au2 | 46774 | 29 | 39 | 1.76 |
| Aa2 | 45796 | 34 | 42 | 1.51 |
| Bc1 | 31171 | 34 | 34 | 1.81 |
| Bu1 | 44242 | 28 | 39 | 1.21 |
| Ba1 | 46998 | 24 | 28 | 1.46 |
| Bb1 | 47974 | 24 | 24 | 1.36 |
| Bc2 | 45265 | 33 | 38 | 1.77 |
| Bu2 | 46229 | 30 | 38 | 1.71 |
| Ba2 | 49722 | 27 | 63 | 1.6 |
| Bb2 | 46055 | 26 | 29 | 1.57 |

**Table S4 Diversity index of each clone library of AOB at different heights and time in TFCW-MFC.**

| Sample | Sequences | OTUs | Chao | Shannon |
| --- | --- | --- | --- | --- |
| Ac1 | 23223 | 150 | 154 | 2.68 |
| Au1 | 23325 | 119 | 129 | 2.76 |
| Aa1 | 17964 | 213 | 283 | 3.35 |
| Ab1 | 34442 | 136 | 146 | 1.42 |
| Ac2 | 27270 | 95 | 133 | 1.26 |
| Au2 | 29385 | 152 | 197 | 2.61 |
| Aa2 | 32886 | 184 | 292 | 2.45 |
| Ab2 | 24621 | 117 | 125 | 2.52 |
| Bu1 | 30923 | 122 | 137 | 2.72 |
| Bb1 | 29490 | 152 | 196 | 3.1 |
| Bc2 | 36900 | 74 | 100 | 1.89 |
| Bu2 | 40518 | 112 | 142 | 1.7 |
| Ba2 | 36330 | 109 | 155 | 2.39 |
| Bb2 | 19992 | 97 | 121 | 2.72 |

**Table S5** Diversity index of each clone library of NOB at different heights and time in TFCW-MFC.

| Sample | Sequences | OTUs | Chao | Shannon |
| --- | --- | --- | --- | --- |
| Ac1 | 44793 | 62 | 64 | 2.01 |
| Au1 | 40901 | 44 | 46 | 2.43 |
| Aa1 | 36090 | 51 | 62 | 2.53 |
| Ab1 | 32378 | 43 | 43 | 2.34 |
| Ac2 | 21523 | 28 | 43 | 1.98 |
| Au2 | 34444 | 48 | 51 | 2.46 |
| Aa2 | 43932 | 37 | 44 | 1.76 |
| Ab2 | 42923 | 28 | 36 | 1.98 |
| Bc1 | 30697 | 27 | 28 | 2.3 |
| Bu1 | 43620 | 42 | 42 | 2.38 |
| Ba1 | 32087 | 30 | 30 | 1.75 |
| Bb1 | 40918 | 29 | 39 | 1.81 |
| Bc2 | 32693 | 31 | 31 | 2.22 |
| Bu2 | 37428 | 40 | 40 | 2.43 |
| Ba2 | 39014 | 31 | 31 | 1.91 |
| Bb2 | 30128 | 29 | 29 | 1.85 |

**Table S6** Diversity index of each clone library of 16S rRNA V4-V5 region at different heights and time in TFCW-MFC.

| Sample | Sequences | OTUs | Chao | Shannon |
| --- | --- | --- | --- | --- |
| Ac1 | 45404 | 1826 | 2249 | 5.75 |
| Au1 | 39091 | 1096 | 1371 | 5.04 |
| Aa1 | 35406 | 1352 | 1608 | 5.45 |
| Ab1 | 29157 | 587 | 811 | 3.56 |
| Ac2 | 33470 | 2225 | 2674 | 6.37 |
| Au2 | 39437 | 1859 | 2529 | 5.58 |
| Aa2 | 27188 | 1626 | 2111 | 5.89 |
| Ab2 | 24689 | 1014 | 1375 | 5.04 |
| Bc1 | 24077 | 2223 | 2766 | 6.63 |
| Bu1 | 23699 | 1983 | 2548 | 5.96 |
| Ba1 | 21675 | 1683 | 2242 | 6.07 |
| Bb1 | 27068 | 1527 | 1984 | 5.58 |
| Bc2 | 18715 | 2042 | 2200 | 6.67 |
| Bu2 | 22863 | 2145 | 2691 | 6.54 |
| Ba2 | 24846 | 2053 | 2649 | 6.26 |
| Bb2 | 19921 | 1643 | 2157 | 5.92 |


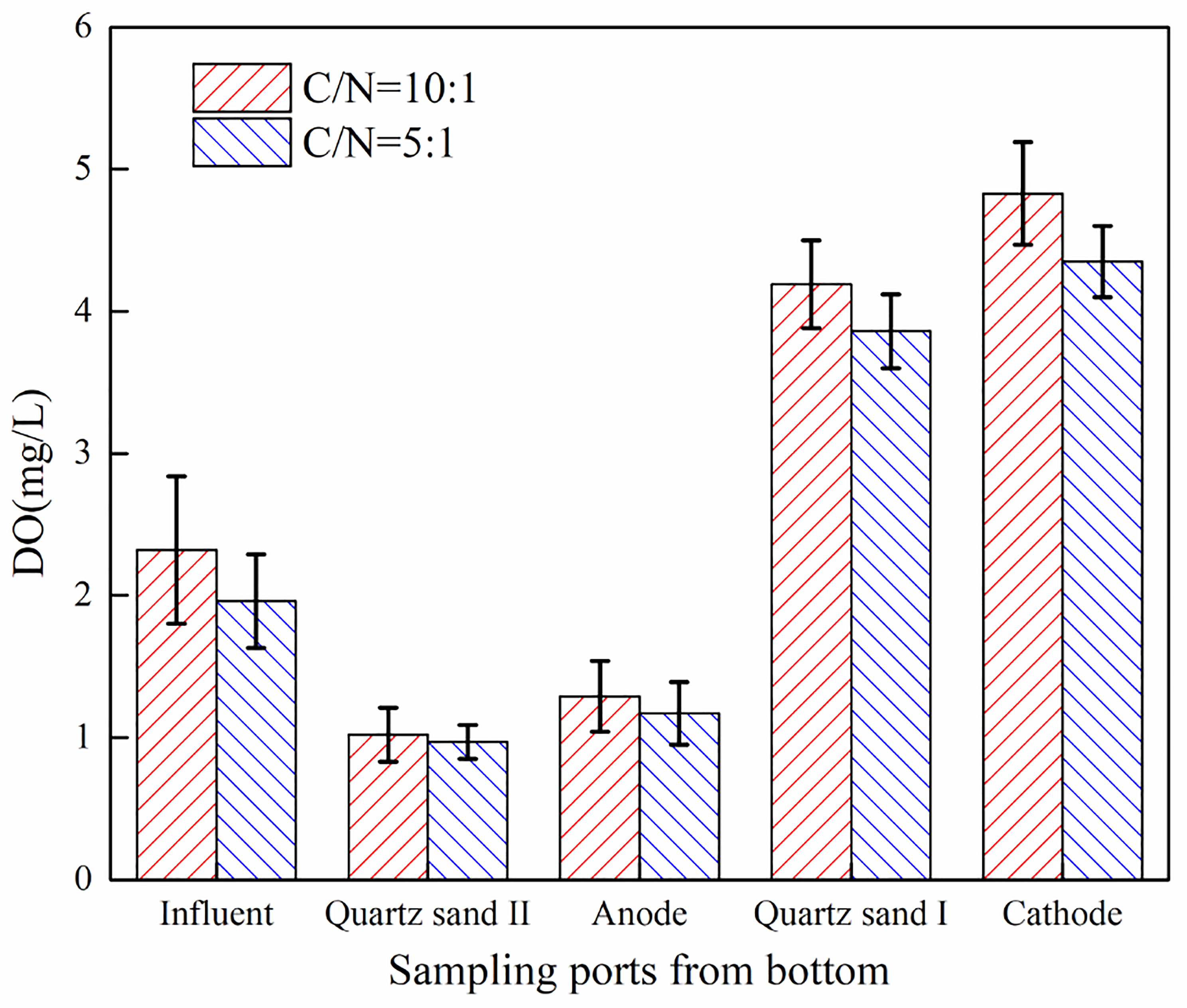


**Fig. S1** DO profile of TFCW-MFC at different C/N ratios.

**Fig. S2** Relative change of shannon indexes at different sampling points of TFCW-MFCs.

| 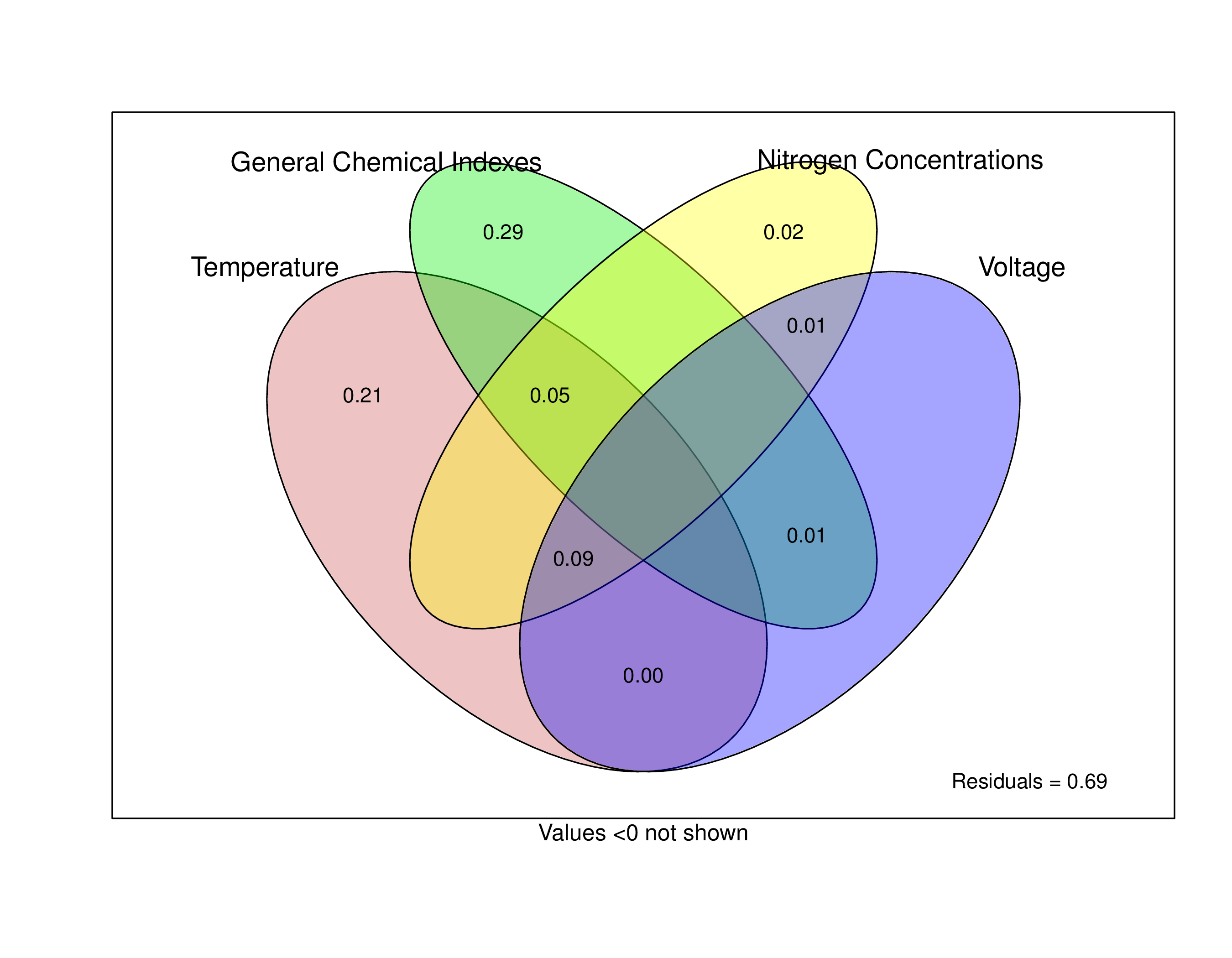  (A) | 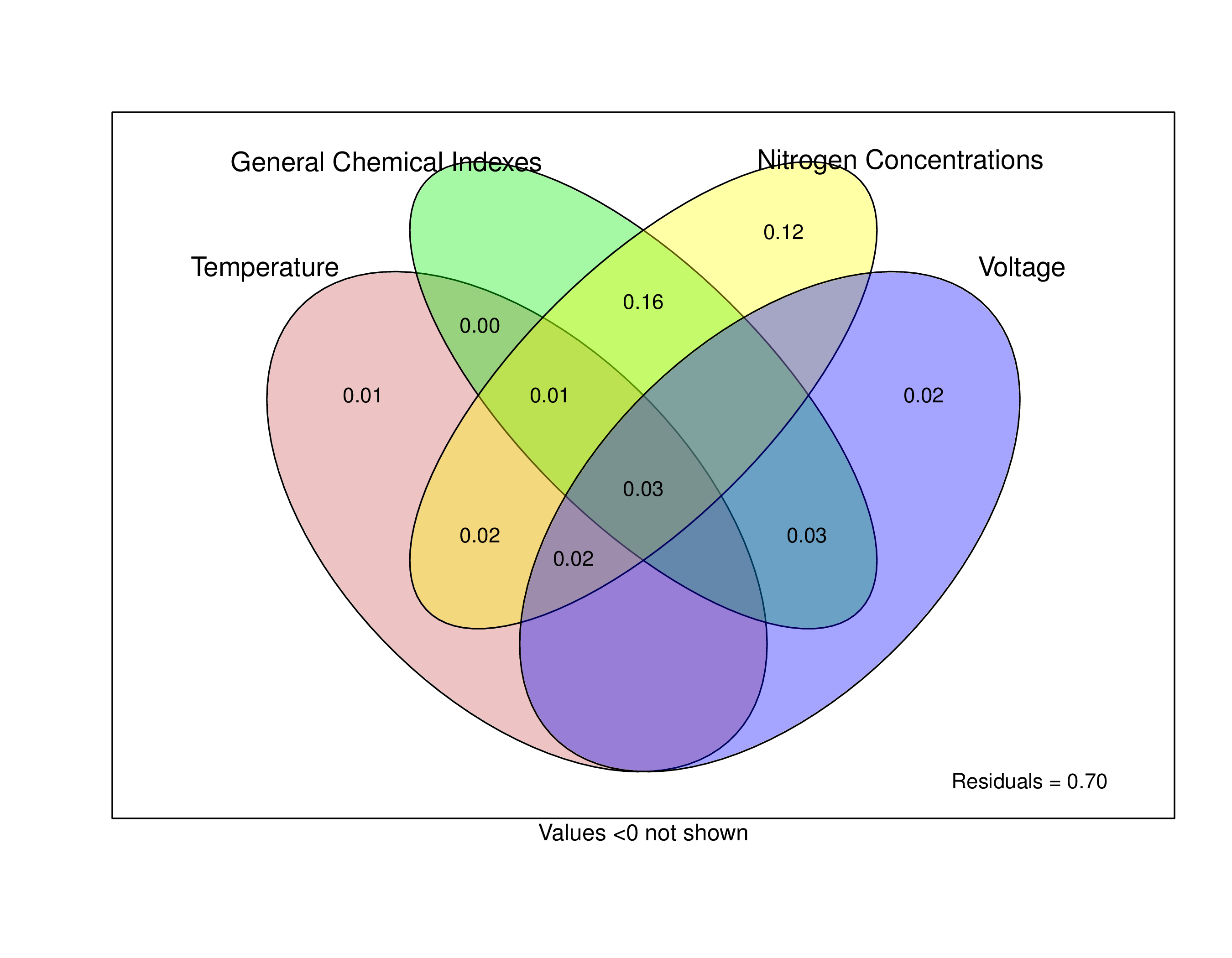  (B) |
| --- | --- |
| 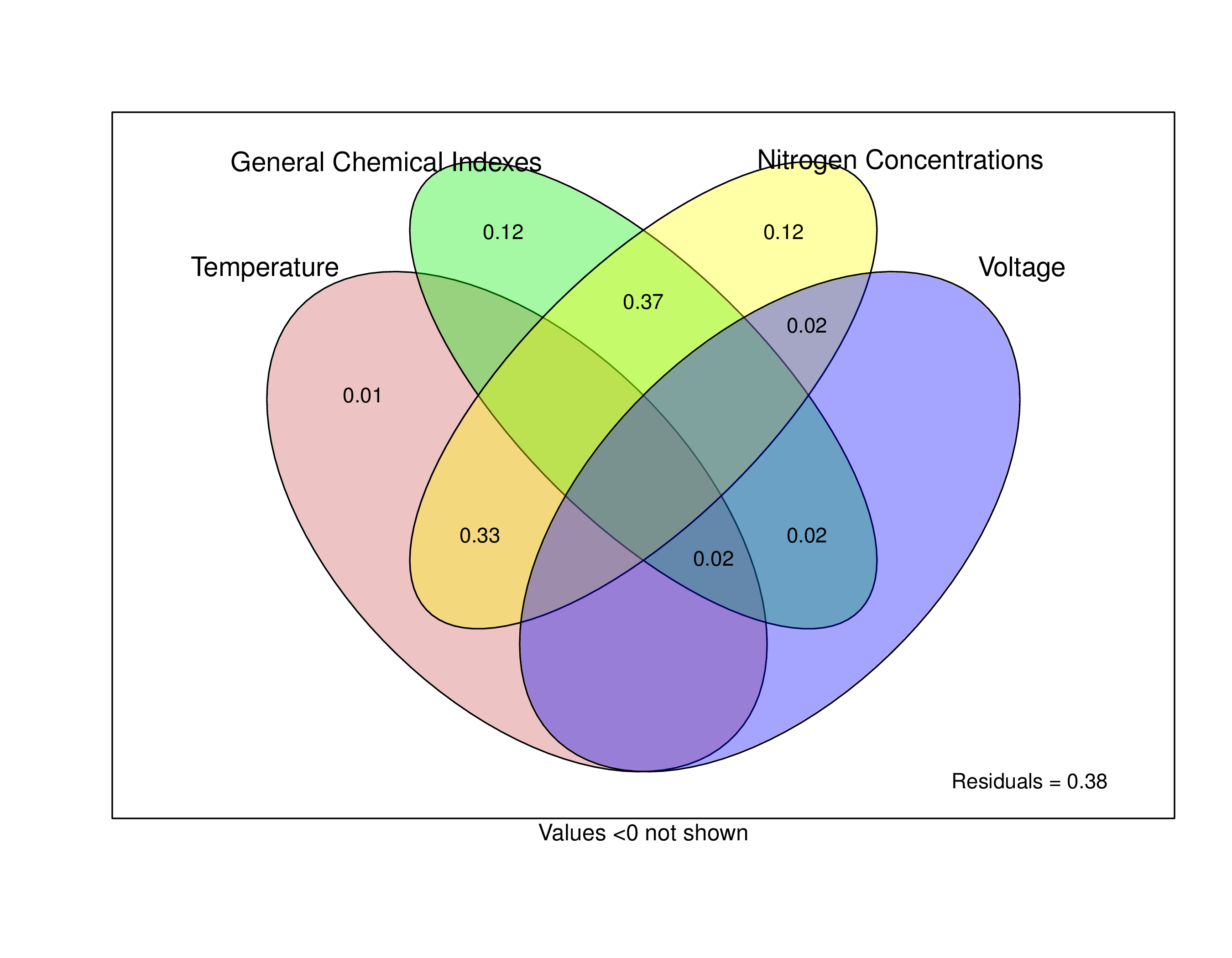  (C) | 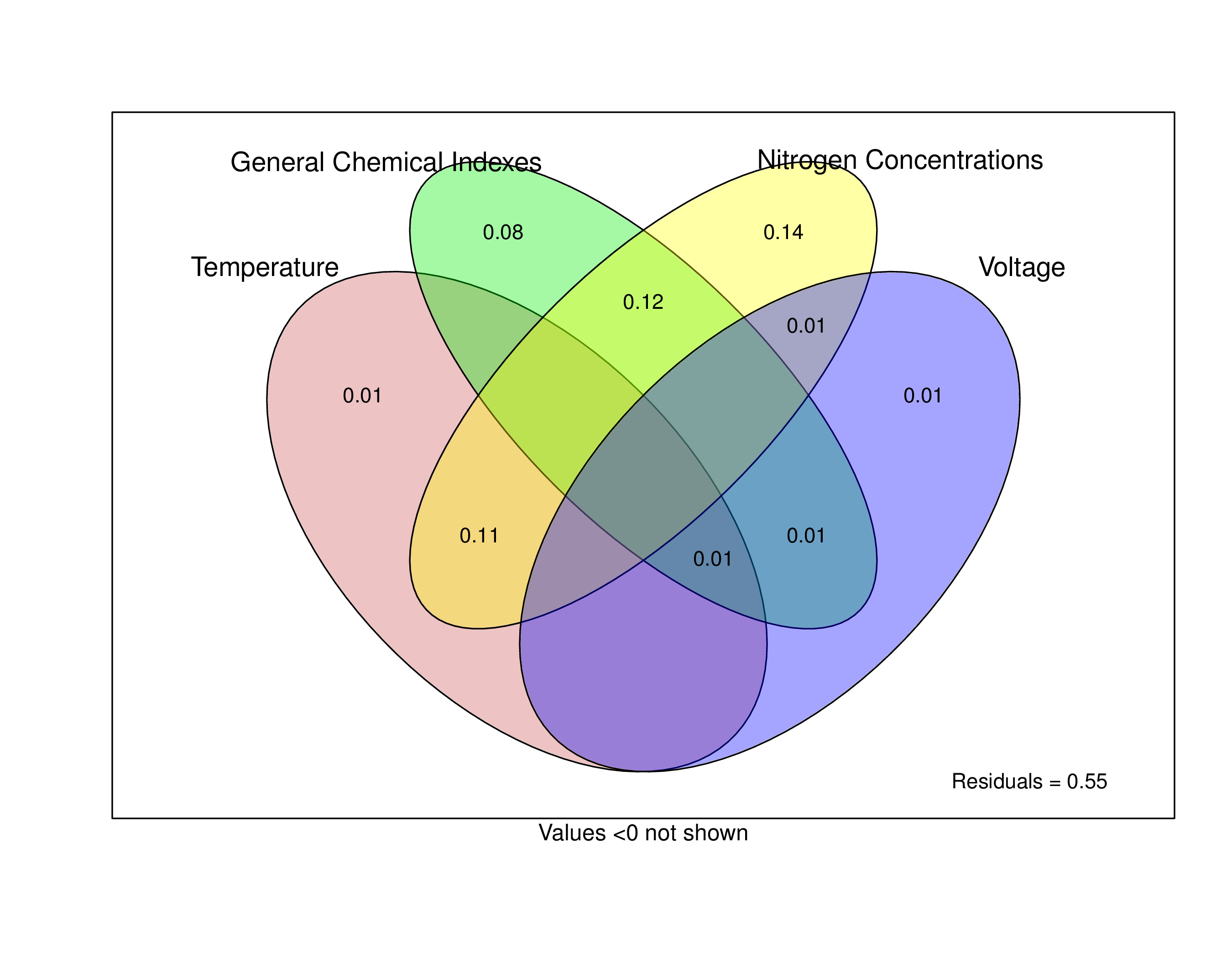  (D) |

**Fig. S3** VPA analysis curves of AOA (A), AOB (B), NOB (C), and 16S rRNA V4-V5 region (D).
